# 825 A Group Comparison of the Use of Autologous Skin Cell Suspension in Geriatric Burn Patients

**DOI:** 10.1093/jbcr/iraf019.356

**Published:** 2025-04-01

**Authors:** Michael Feldman, Megan Newsom, Prabhu Senthil-Kumar

**Affiliations:** Virginia Commonwealth University; Virginia Commonwealth University; Evans-Haynes Burn Center

## Abstract

**Introduction:**

Burn injuries significantly impact patients’ lives through pain, scarring, altered quality of life, and financial strain. There is a clear link between increasing age and mortality with burn patients. In addition, elderly burn patients are known to have a higher incidence of co-morbidities placing them at risk for worse outcomes.

Autologous skin cell suspension is a method for reconstructing burn wounds that allows for an 80:1 expansion of skin cells. This has been proven to reduce the donor site burden allowing wound closure with less skin.

The purpose of this study is to review our outcomes using autologous skin cell suspension in the Geriatric population.

**Methods:**

This study was awarded exempt status by our IRB. Data was pulled from the burn registry for all burn patients aged 65 years and greater from January 2020 until October 2023. This included demographics, presence of minor complications, major complications, readmission within 30 days, time to healing, disposition, and length of stay. Patients who underwent treatment with autologous skin cell suspension were identified and compared to age, TBSA, comorbidity, and gender matched controls. Results compared complications, readmission within 30 days, time to healing (days), disposition and length of stay. We are continuing the review process and have added patients through July 2024 which is ongoing.

**Results:**

Our review identified 40 patients with 20 in the ASCS group and 20 in the control group. There were no significant differences between the ASCS and Non-ASCS groups. Analysis was performed using the Wilcox rank sum for time to healing and Fisher’s exact to analyze 95% re-epithelialization and disposition. Results include any complication, P = 0.5006, minor complications, P = 0.7164, major complications, P = 1, and readmission within 30 days, P = 0.4872.

Time to healing showed a trend towards quicker healing in the ASCS group but did not meet statistical significance (time to healing in days, P = 0.0675, ASCS group: mean 21.05, SD 17.88 versus Non-ASCS group mean 28.42, SD 17.44). There was no difference in 95% re-epithelialization at 4 weeks, P = 0.3008.

ASCS and Non-ASCS patients had similar routes of disposition, P = 0.3235 and length of stay, P = 0.2664 (ASCS group: mean 23.6, SD 15.39 versus Non-ASCS group: mean 22.85, SD 32.09).

**Conclusions:**

The Geriatric population remains vulnerable to morbidity and mortality from burn injuries. We sought to characterize the use of ASCS in this population. While our data does not support a statistical difference between the groups there is a trend towards earlier healing in the ASCS group. We continue to collect analyze additional data which will be available for presentation at the ABA meeting.

**Applicability of Research to Practice:**

This research is paramount of understanding the role of autologous skin cell suspension in treating geriatric burn patients.

**Funding for the Study:**

N/A

